# Clinical and imaging findings associated with preservation of knee joint health over 8 years in individuals aged 65 and over: data from the OAI

**DOI:** 10.1186/s12891-024-07590-z

**Published:** 2024-06-26

**Authors:** Felix G. Gassert, Gabby B. Joseph, John A. Lynch, Johanna Luitjens, Michael C. Nevitt, Charles E. McCulloch, Nancy E. Lane, Sharmila Majumdar, Thomas M. Link

**Affiliations:** 1 Department of Radiology and Biomedical Imaging, University of California, San Francisco, 185 Berry Street, Lobby 6, Suite 350, San Francisco, CA 94107 USA; 2 Department of Radiology, Klinikum rechts der Isar, Technical University of Munich, Ismaninger Str. 22, 81675 Munich, Germany; 3 Department of Epidemiology and Biostatistics, University of California, San Francisco, CA USA; 4 Center for Musculoskeletal Health, Department of Medicine, University of California, Davis, CA USA

**Keywords:** Osteoarthritis, Magnetic resonance imaging, Older individuals, Protective factors

## Abstract

**Objective:**

While risk factors for osteoarthritis (OA) are well known, it is not well understood why certain individuals maintain high mobility and joint health throughout their life while others demonstrate OA at older ages. The purpose of this study was to assess which demographic, clinical and MRI quantitative and semi-quantitative factors are associated with preserving healthy knees in older individuals.

**Methods:**

This study analyzed data from the OA Initiative (OAI) cohort of individuals at the age of 65 years or above. Participants without OA at baseline (BL) (Kellgren-Lawrence (KL) ≤ 1) were followed and classified as incident cases (KL ≥ 2 during follow-up; *n* = 115) and as non-incident (KL ≤ 1 over 96-month; *n* = 391). Associations between the predictor-variables sex, age, BMI, race, clinical scoring systems, T_2_ relaxation times and Whole-Organ Magnetic Resonance Imaging-Score (WORMS) readings at BL and the preservation of healthy knees (KL ≤ 1) during a 96-month follow-up period were assessed using logistic regression models.

**Results:**

Obesity and presence of pain showed a significant inverse association with maintaining radiographically normal joints in patients aged 65 and above. T_2_ relaxation times of the lateral femur and tibia as well as the medial femur were also significantly associated with maintaining radiographically normal knee joints. Additionally, absence of lesions of the lateral meniscus and absence of cartilage lesions in the medial and patellofemoral compartments were significantly associated with maintaining healthy knee joints.

**Conclusion:**

Overall, this study provides protective clinical parameters as well as quantitative and semi-quantitative MR-imaging parameters associated with maintaining radiographically normal knee joints in an older population over 8 years.

## Significance and innovations

Most protective parameters for osteoarthritis seem to be similar in the elderly as compared to mixed age cohorts. Nevertheless, some risk factors may be different in the elderly, especially regarding quantitative imaging parameters.

## Introduction

Osteoarthritis (OA) is the most common form of arthritis and a major cause of physical disability and reduced quality of life in the elderly [1]. OA is most commonly located in the knee with an estimated prevalence of 27% at the age of 70 [2]. With an aging population, the economic burden and the loss in quality of life due to OA is expected to strongly increase within the next decades [3].

There are well known risk factors for OA in the general population. Nevertheless, it is unclear why certain individuals maintain high mobility and joint health at older ages while other individuals demonstrate cartilage breakdown and OA. The characteristics of patients above of the age of 65 who have and maintain radiographically normal joints have not been well investigated. On the other hand, there is some evidence suggesting that individuals who develop OA at older ages have different risk factors than those of younger age groups. For example, Driban et al. have shown that obese patients and patients at older age are at high risk of developing accelerated OA [4]. In addition, studies have demonstrated that physical activity, waist circumference and pain impact physical function or quality of life in older patients with OA [5–7].

Multiple of these studies are based on the Osteoarthritis Initiative (OAI), a longitudinal, multi-center cohort study that recruited 4796 individuals and is sponsored by the US National Institutes of Health (NIH) including clinical and imaging parameters during up to 8 years (OAI, https://oai.nih.gov).

In general, most studies on knee OA have focused on using radiographs with Kellgren-Lawrence (KL) scoring to define OA [1]. Nevertheless, MR imaging has been shown to give a more comprehensive understanding of structural OA development. The MR based Whole-Organ Magnetic Resonance Imaging Score (WORMS) of the knee provides a reliable multi-feature assessment tool in OA of the knee [8, 9]. Besides more granular analysis of meniscal, cartilage and bone marrow edema like lesions, MR imaging helps evaluate effusion and synovitis in the knee – an important mediator of OA [10]. Moreover, recent studies revealed associations between cartilage T_2_ relaxation times determined on MR images and the onset of cartilage lesions [11, 12], indicating the potential of MR imaging parameters to possibly predict morphologic OA. To date, however, there is only a limited number of studies investigating MRI findings in older patients with OA or risk factors for OA. One study, for example, has shown that full-thickness cartilage defects determined on MR images are an important predictive factor for the progression of OA to a total knee replacement in older patients [13]. A study by Sharma et al. in a mixed age group showed that worsening MRI lesions status was associated with concurrent incident radiographic OA and therefore proposed that these lesions represent early OA [14].

Although some studies evaluated MR imaging criteria in OA and others focused on the correlation of clinical parameters and OA in patients of different age groups, so far it is unclear which factors help preserve healthy knees at higher ages and no study has been performed a combined investigation of demographic, clinical and imaging characteristics of individuals older than 65 years who maintain radiographically normal knees. In this study, we therefore aim (i) to analyze a cohort of participants from the OAI cohort with KL 0 and 1 knee radiographs over 8 years concerning demographic, clinical factors, and MRI quantitative and semi-quantitative parameters, which will serve as predictors and (ii) to compare this cohort with an age-matched cohort that develops radiographic OA above the age of 65 over 8 years.

The purpose of this study was to provide a comprehensive understanding of characteristics of knee joints in older individuals who maintain radiographically normal morphology over 8 years and the specific protective factors in this age group.

## Patients and methods

### Participant selection

The analyses in this study are based on data from the OAI (https://nda.nih.gov/oai), a longitudinal, observational multi-center study with a cohort size of *n* = 4796 individuals, designed to assess biomarkers in OA. This dataset includes clinical information with a symptom questionnaire and MRIs of both knees obtained at baseline (BL), 12-, 24, 36-, 48- 72- and 96-month follow-up. Institutional review boards of each center approved informed consent documentation, study protocols and amendments. All investigations were carried out in compliance with the Helsinki Declaration.

**Fig. 1:**
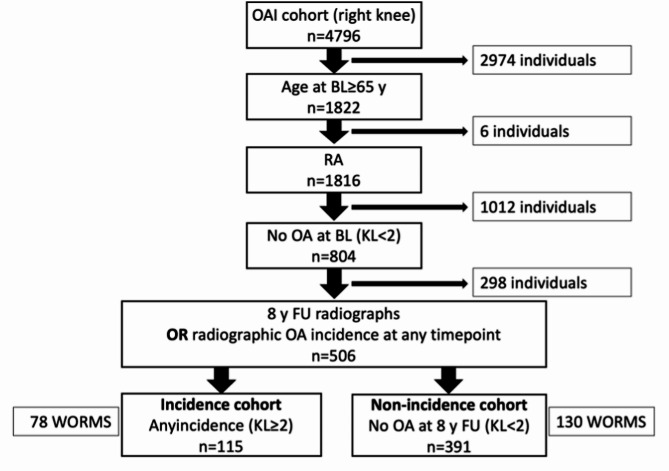
Flow chart illustrating the inclusion and exclusion criteria for participant selection. Participants needed either an 8 year follow up visit without radiographic OA or radiographic incidence of OA at any other timepoint (then 8 year follow up radiograph was not required as OA is assumed to be irreversible). BL = Baseline; FU = Follow up; KL = Kellgren Lawrence; n = Number; OAI = Osteoarthritis Initiative; RA = Rheumatoid Arthritis; WORMS = Whole-Organ Magnetic Resonance Imaging Score; y = years

Figure 1 shows a flow chart illustrating the inclusion and exclusion criteria for this analysis. Our analysis focused on the right knee only as the full imaging complement was available for the right knee including T_2_ relaxation time measurements. From the 4796 participants in the OAI 1822 were at least 65 years of age at the baseline visit. Six participants were excluded due to rheumatoid arthritis at BL. In order to analyze features associated with the onset of OA, we excluded participants with radiographic OA at BL, which was defined as a KL score ≥ 2 as reported previously [15]. The remaining participants without OA at BL were classified into two outcome groups: A control group and an incidence group. Control group individuals were defined as those with a 96-month follow-up visit without OA demonstrated on knee radiographs (*n* = 391). The incidence group consisted of those individuals who showed OA (KL > 1) at any follow-up visit (*n* = 115).

### Image acquisition

MR images were acquired at four centers (Columbus, Ohio; Baltimore, Maryland; Pittsburgh, Pennsylvania and Pawtucket, Rhode Island), using four identical 3.0 Tesla scanners (Siemens Magnetom Trio, Erlangen, Germany). Acquired sequences of the knee used in this study included: (i) coronal 2D intermediate-weighted (IW) turbo spin-echo (TSE) [repetition time (TR) / echo time (TE); spatial resolution; field of view (FOV); slice thickness; gap] [3700 ms / 29 ms; 0.365 mm x 0.456 mm; 140 mm; 3.0 mm; 0 mm), (ii) sagittal, fat-saturated (FS) 2D IW TSE [3200 ms / 30 ms; 0.357 mm x 0.511 mm; 160 mm; 3 mm; 0 mm), (iii) coronal 3D fast low angle shot with water excitation (FLASH WE) [7.57 ms / 20 ms; 0.313 mm x 0.313 mm; 160 mm; 1.5 mm; 0 mm] and (iv) sagittal 3D dual-echo steady state sequence with water excitation (DESS WE) [4.7 ms / 16.3 ms; 0.365 mm x 0.456 mm; 140 mm; 1.5 mm; 0 mm] with axial and coronal reformations. To allow quantitative assessment of cartilage T_2_ relaxation times, a sagittal 2D multi slice multi echo sequence (MSME) was also included [2700 ms / 10–70 ms; 0.313 mm x 0.446 mm; 120 mm; 3.0 mm / 0.5 mm]. Detailed information on imaging protocols is available online (https://oai.epi-ucsf.org/datarelease/operationsManuals/MRI_ManualRev.pdf) [16].

### Clinical parameters

Influence of sex, age, BMI (normal vs. obese (≥ 30 kg/body height in m^2^)) and race (white vs. non-white) on the onset of OA were analyzed. Furthermore, the clinical scoring system “Western Ontario and McMaster Universities Osteoarthritis” (WOMAC) with subscales for stiffness, pain, and activity of daily life were included in the analysis as reported previously [17] [18]. , with scores ranging from 0 to 96 for the total WOMAC where 0 represents the best health status and 96 the worst possible status. Furthermore, the Physical Activity Scale for the Elderly (PASE), a scoring instrument that measures the level of physical activity in individuals aged 65 years and older on a scale of -400 to + 400 was evaluated [19].

### Image analysis

Image analysis was performed on picture archiving communication system workstations (Agfa, Ridgefield Park, NJ, USA). Structural degenerative joint disease was semi-quantitatively graded for each exam using a modified version of the Whole-Organ Magnetic Resonance Imaging Score (WORMS) system in all participants, as previously described [8, 9]. Accordingly, cartilage lesions were graded in six locations (at the patella, trochlea, medial and lateral femoral condyle, medial and lateral tibial plateau, respectively). Meniscal lesions were also graded in six locations (anterior horn, body, and posterior horn, for medial and lateral meniscus, respectively). WORMS readings at baseline were available in a subset of participants: in 130 participants of the control group and in 78 participants of the incidence group. Intra-class correlation coefficients (ICC) demonstrating excellent inter- and intra-reader reproducibility for modified WORMS gradings of cartilage and menisci have previously been reported by our group (ICCinter-reader = 0.95–0.97 and ICCintra-reader = 0.97–0.98 respectively) [9].

Additionally, cartilage T_2_-relaxation times were included in this analysis. As reported previously, we developed a fully automatic method for reliable cartilage segmentation of knee MRI volumes on a T_2_ mapping sequence [11, 20, 21]. This algorithm was applied to all MR scans in the OAI dataset. The predicted cartilage compartments were then fully automatically subsegmented into lateral tibia (LT), medial tibia (MT), central lateral femur (cLF), central medial femur (cMF) and patella (P) compartments as reported previously [21]. The average T_2_-relaxation times in those regions were defined.

### Selection of primary predictors

To reduce probability of error due to multiple testing, the predictor variables have been separated into primary, and secondary categories based on their importance for the proposed research and based on the preliminary data and previous research. As the predictor variables BMI and the WOMAC pain score are well established risk factors for OA in the general population, these clinical predictor variables were chosen as primary predictors [22, 23]. Age served as secondary predictor, as this study was performed in a subgroup of the OAI at older ages already. Race, due to the relatively small sample size, and the remaining clinical (sub-)scores (i.e. WOMAC stiffness) also served as secondary predictors as there is less evidence for association with OA incidence. From the semiquantitative imaging parameters, the sum score over all cartilage lesions was used as primary predictor as cartilage lesions have been shown to be associated with incidence of OA in a mixed age group [14]. The sum score over the lateral and medial meniscus as well as the cartilage lesions in the lateral, the medial, and the patellofemoral compartment were used as secondary predictors. From the quantitative imaging parameters, the mean T_2_ value over all regions was designated as a primary predictor, as it provides information on all compartments. Consequently, the T_2_ values of the lateral/medial femur/tibia and the patella were designated as secondary predictors.

### Statistical analysis

All statistical analyses were performed using the statistical package R version 3.2.4 (R Foundation for Statistical Computing, Vienna, Austria), with a two-sided level of significance of α = 0.05. Descriptive statistics for participant age and sex at baseline were analyzed using crosstabs for sex and means and standard deviation (SD) for age. The outcome variable was a binary variable defined by whether an older individual developed radiographic OA within 96 months after the BL scan (yes/no). Associations between the predictor-variables at baseline (age, sex, BMI, race, WOMAC pain/total, PASE, WORMS, T_2_-values) and the onset of radiographic OA (yes/no) were assessed using logistic regression models and outcomes reported as odds ratios (OR) for developing OA during this timeframe. ORs are reported per standard deviation change of each predictor (labeled as *sOR* in the results section). Age, sex, and BMI adjustments were included in all analysis.

## Results

### Participants demographics

Demographics are shown in Table 1. Overall, 506 participants were included in the analysis (115 older individuals with incident radiographic OA, and 391 without incident radiographic OA). Mean time before radiographic onset of OA was at 3.97 years with 34/20/18/9/30/14 patients showing onset of OA at 12/24/36/48/72/96-month follow-up. Mean age was at 70.3 ± 4.1 years for the incidence group and 70.5 ± 3.8 years for the non-incidence group with no significant difference between groups (*p* = 0.70). Also, no significant difference in sex was observed for the two groups (206 females in the incidence group (52.7%); 68 females in the non-incidence group (59.1%); *p* = 0.17). There was no significant difference between the average BMI of the incidence group at 28.22 [24.5-31.94] and the non-incidence group at 26.67 [23.0-30.34] (*p* = 0.21).

**Table 1:** Patient demographics at baseline

	Incidence (*n* = 115)	Non-Incidence (*n* = 391)	all (*n* = 506)	*p*-value
Sex				
Women	68	206	274	0.17
Men	47	185	232	
Age [years]	70.3 ± 4.1	70.5 ± 3.8	70.4 ± 3.8	0.7
BMI	28.2 ± 3.7	26.7 ± 3.7	27.02 ± 3.7	0.21

### Primary predictors

Associations between primary predictor variables and maintaining radiographically normal joints are reported in Table 2. Obesity was significantly associated with a lower OR (per SD change in the predictor) of maintaining radiographically normal joints (standardized OR (sOR): 0.43, [95% CI = 0.23–0.79], *p* = 0.007). The pain-subscale for the WOMAC scoring system also was significantly associated with the OR of maintaining radiographically normal joints (sOR WOMAC pain: 0.69, [0.56–0.85], *p* < 0.001). Additionally, the overall sum score for all cartilage regions derived from the WORMS readings as well as average T_2_ values of all compartments of the knee were significantly associated with the OR of maintaining radiographically normal joints (sOR: 0.62, [0.46–0.82], *p* = 0.001; sOR: 0.72, [0.58–0.82], *p* = 0.003).

**Table 2:** Standardized odds ratios of primary predictors on maintaining radiographically normal joints

Parameter	sOR	s95%-CI	*p*-value
BMI ≥ 30	**0.43**	**0.23–0.79**	0.007**
WOMAC Pain	**0.69**	**0.56–0.85**	< 0.001**
Average T_2_	**0.72**	**0.58–0.82**	0.003**
WORMS Cart. Lesions sum	**0.62**	**0.46–0.82**	0.001**

Standardized OR (sOR) refers to the OR of an increase by one standard deviation of the predictor. *P* values are given for individual models. All values were adjusted for age, sex, and BMI adjustments were included in all analysis. BMI = body mass index; CI = confidence interval; OR = odds ratio; s = standardized; sum = sum score; WOMAC = Whole-Organ Magnetic Resonance Imaging Score; **: *p* < 0.01

### Secondary predictors

Associations between secondary predictor variables and maintaining radiographically normal joints are reported in Table 3. There was no significant association observed between sex, age, race and maintaining radiographically normal joints (*p* = 0.14/0.58/0.53). The total score of the clinical scoring system WOMAC significantly decreased the OR of maintaining radiographically normal joints (sOR: 0.68, [0.56–0.83], *p* = 0.001). Furthermore, there was no significant association between PASE score at baseline and the development of incident OA throughout an 8-year follow-up period (*p* = 0.97).

**Table 3:** Standardized odds ratios of secondary clinical, semiquantitative (WORMS) and quantitative predictors on maintaining radiographically normal joints

	Parameter	sOR	95%-CI	*p*-value
Clinical	Sex	0.72	0.47–1.1	0.136
	Age	1.06	0.86–1.32	0.581
	Race (if non-white)	1.32	0.59–3.33	0.534
	**WOMAC Total**	**0.68**	**0.56–0.83**	< 0.001**
	PASE	1.00	0.81–1.24	0.972
WORMS	**Lat. Meniscus sum**	**0.61**	**0.45–0.81**	0.001**
	Med. Meniscus sum	0.87	0.66–1.15	0.324
	Cart. lesions lat. comp.	0.85	0.65–1.11	0.244
	**Cart. lesions med. comp.**	**0.71**	**0.54–0.93**	0.016*
	**Cart. lesions pat-fem.**	**0.67**	**0.51–0.88**	0.004**
Quantitative	**T**_**2**_ **lat. Tibia**	**0.76**	**0.62–0.94**	0.014**
	**T**_**2**_ **lat. Femur**	**0.7**	**0.56–0.86**	0.001**
	T_2_ med. Tibia	0.81	0.66–1.01	0.069
	**T**_**2**_ **med. Femur**	**0.68**	**0.54–0.84**	0.001*
	T_2_ Patella	0.96	0.79–1.19	0.734

Standardized OR refers to the OR of an increase by one standard deviation. *P* values are given for individual models. All values were adjusted for age, sex, and BMI adjustments were included in all analysis. ADL = activities of daily life; BMI = body mass index; CI = confidence interval; lat = lateral; med = medial; OR = odds ratio; s = standardized; sum = sumscore for the different regions; PASE = Physical Activity Scale for the Elderly; QoL = quality of life; WOMAC = Whole-Organ Magnetic Resonance Imaging Score; WORMS = Whole-Organ Magnetic Resonance Imaging Score; *: *p* < 0.05; **: *p* < 0.01

Regarding the WORMS readings, an elevated sum score for the lateral meniscus significantly decreased the OR for maintaining radiographically normal joints (sOR: 0.61, [0.45–0.81], *p* = 0.001), whereas the sum score of the medial meniscus did not show a significant association (*p* = 0.27). Regarding cartilage lesions, the sum score of the medial compartment (medial femur and tibia) and the patellofemoral compartment were significantly associated with a lower OR for maintaining radiographically normal joints (sOR medial compartment: 0.71, [0.54–0.93], *p* = 0.016; sOR patellofemoral compartment 0.67, [0.51–0.88], *p* = 0.004), whereas the sum score for the lateral compartment did not show any significant associations (*p* = 0.29).

Cartilage T_2_ values in both, the lateral femur and tibia showed a significant association with maintaining radiographically normal joints (sOR lateral tibia: 0.76 [0.62–0.94], *p* = 0.014; lateral femur: 0.7, [0.56–0.86], *p* = 0.001). Also, elevated cartilage T_2_ values in of the medial femur were significantly associated with lower odds of maintaining radiographically normal joints (sOR: 0.68, [0.54–0.84], *p* = 0.001). No significant associations between T_2_ values in the patella cartilage and incident OA were observed (*p* = 0.73).

## Discussion

This study assessed the associations between demographic, clinical and imaging findings including WORMS readings (predictors) with maintaining radiographically normal knee joints (outcome) in OAI participants 65 years and above. Obesity, pain, functional impairment as well as high cartilage T_2_ relaxation times in the lateral compartment and of the medial femur significantly decreased the OR for maintaining radiographically normal joints. Moreover, WORMS readings demonstrated significant inverse associations between the sum scores for the lateral meniscus as well as the cartilage of the medial and the patellofemoral compartment and maintaining healthy knee joints.

It is well known that older people show a different set of risk factors for multiple diseases as compared to a younger population, especially in neurological diseases [24]. Comparable to younger participants, obesity increased the risk of developing incident radiographic OA, which is in line with a study by Driban et al. [4]. Although, female sex is a well-established risk factor for OA in the general population, results in the older participants of this study were not significant [22]. A lower WOMAC pain score as well as WOMAC total score were significantly associated with maintaining radiographically normal knee joints in the older group. Accordingly, previous studies showed a correlation of pain with the incidence of OA using cartilage volume loss and incident radiographic knee OA as outcomes [23, 25–28].

Edd et al. examined the longitudinal changes of knee cartilage T_2_ relaxation times and reported increases in T_2_ relaxation times of the medial compartment of the knee during radiographic progression of OA [29]. Liebl et al. showed in a middle-aged subgroup of the OAI (mean age 59 years), that early T_2_ changes predict the onset of radiographic knee OA [30]. Although they observed that effect in the entire lateral compartment and the medial femur, similar to our study, they did not observe a significant effect for the medial tibia. Nevertheless, different from our results, in their study, a highly significant correlation of the T_2_ relaxation times of the patella with the onset of OA was found using individual linear regression models. Heilmeier et al. showed that increased T_2_ relaxation times of the cartilage in the lateral compartment significantly increased the risk of total knee arthroplasty within 4–7 years [31]. This allows the conclusion that T_2_ values of the lateral compartment may be an important predictor of OA progression in all age groups, whereas the cartilage of the patella may be more important in younger patients as compared to older patients in maintaining healthy knees.

The WORMS system used in this study provides a multi-feature, whole-organ assessment of the knee in OA using conventional MR images [8]. The absence of focal lesions of the patellofemoral cartilage was a significant predictor for maintaining healthy knees in this older participant group. Cartilage lesions in the medial compartment were associated with developing OA, whereas lesions in the lateral compartment did not show such an association. Hafezi-Nejad et al. also demonstrated a significant increase in the hazard ratio for future knee replacement through increased cartilage lesions scores of WORMS in a subset of the OAI including all age-groups [32]. Sharma et a. showed in a mixed age group that worsening MRI lesions status was associated with concurrent incident radiographic OA. Nevertheless, these studies were performed in a mixed age group and did not examine the individual knee compartments separately. Comparable to our results, a study by Yang et al. based on 88 patients found that the medial compartment and the patellofemoral joint degenerate more severely in early stage knee OA [33]. Interestingly, absence of lesions of the lateral meniscus and not the medial meniscus was associated with maintaining healthy knees in the elderly. These results are in line with a study by Badlani et al. in a younger cohort, who also found that lesions of the medial but not the lateral meniscus are more frequent in patients who develop OA as compared to those who maintain healthy knees [34].

This study has some limitations: Firstly, radiographic KL scores are used as outcome measurements, although studies have shown that KL does not fully correlate with disease severity and alternative endpoints have been proposed for OA [35, 36]. Nevertheless, KL is still the most commonly used grading system for OA. Secondly, although the analyzed group of 506 OAI participants and 208 WORMS readings was relatively large as compared to previous studies, some associations remained borderline significant. Studies investigating a larger cohort may resolve this limitation. A further limitation involves the nature of the study design: The study examined statistically significant associations of clinical parameters, imaging parameters and WORMS readings with the development of OA. This does not allow for conclusions on causal relationships or generate concrete clinical implications. Lastly, no head-to-head comparison between older and younger patients has been performed as this was beyond the scope of this study. Overall, further studies are needed to overcome these limitations and confirm our observations, especially regarding a head-to-head comparison of risk factors for OA in different age groups.

In summary, this study describes significant protective factors for maintaining radiographically normal knee joints in an older population, including clinical parameters as well as quantitative and semi-quantitative MR-imaging parameters. Although results of this study suggest that most protective parameters seem to be similar in the elderly as compared to mixed age cohorts, some risk factors may be different in the elderly, especially regarding secondary quantitative parameters of the patella cartilage.

## Data Availability

The underlying data is publicly available (OAI, https://oai.nih.gov).
